# Fatal attraction: flowers lure pollinators as prey in the carnivorous *Drosera hookeri* (Droseraceae)

**DOI:** 10.1093/aob/mcag170

**Published:** 2026-06-22

**Authors:** Caroline L Gross, Joshua D Whitehead, Wendy A Waters, K David Mackay, Ellis S G Mackay, Camila Silveira Souza

**Affiliations:** Ecosystem Management, University of New England, Armidale, NSW 2351, Australia; Ecosystem Management, University of New England, Armidale, NSW 2351, Australia; Ecosystem Management, University of New England, Armidale, NSW 2351, Australia; Ecosystem Management, University of New England, Armidale, NSW 2351, Australia; Ecosystem Management, University of New England, Armidale, NSW 2351, Australia; Melbourne Veterinary School, School of Chemistry, Faculty of Science, University of Melbourne, Melbourne, VIC 3010, Australia; Ecosystem Management, University of New England, Armidale, NSW 2351, Australia; Programa de Pós-Graduação em Recursos Naturais, Universidade Federal de Mato Grosso do Sul, Campo Grande, MS, 79070-900, Brazil; Laboratório de Ecologia Vegetal, Programa de Pós-Graduação em Botânica Aplicada, Departamento de Biologia Geral, Universidade Estadual de Montes Claros, Montes Claros, MG, 39401-089, Brazil

**Keywords:** Breeding system, *Drosera hookeri*, drought conditions, floral ecology, leaf traps, pollinator–prey conflict, self-compatibility

## Abstract

**Background and Aims:**

Pollinator–prey conflict can occur in carnivorous plants when they ensnare pollinators in their trap leaves. Although spatial or cue separation often abates this conflict, this study investigates *Drosera hookeri*, which produces flowers in close proximity to leaf traps. We evaluate pollinator–prey conflict and test the hypothesis that flowers facilitate pollinator capture.

**Methods:**

Field studies were conducted between 2007 and 2021 in eastern Australia; the extended duration was necessary because drought conditions often caused sparse flowering or prevented plant emergence. Pollinator diversity and visitation were assessed using video monitoring, and pan traps estimated community abundance. Hand-pollination experiments determined pollinator dependence and pollen limitation. Arthropod abundance in trap leaves was measured before, during and after flowering, and a paired experiment compared prey capture between plants with and without flowers.

**Results:**

*Drosera hookeri* is highly autofertile and self-compatible, with weak pollen limitation in field conditions. We observed a pollinator–prey conflict: pollinators (predominantly *Melangyna viridiceps*) accounted for 57 % of identified prey, indicating that flowers and nearby trap leaves overlap in their effects. Pollinators were not attracted to plants lacking flowers. In flowering plants, pollinators were occasionally tipped into traps when flimsy pedicels (hypothesised to respond to water stress) bent under their weight.

**Conclusions:**

*Drosera hookeri* exhibits a pollinator–prey conflict in a resource-limited system. Pollinator capture appears to result from foraging behaviour and floral proximity rather than direct attraction to traps, contrasting with suggestions that pollinators avoid traps owing to ‘intelligence’ or that *Drosera* mucilage is optimized for smaller prey. Its impact is likely to be asymmetric, with stronger potential effects on male function through loss of pollen vectors, whereas female function is buffered by autonomous selfing.

## Teaser

Carnivorous plants are thought to avoid trapping their own pollinators by separating flowers from traps. We tested the opposite condition in the sundew *Drosera hookeri*, whose flowers occur beside sticky trap leaves. Over 14 years, we found that pollinators are frequently captured after slipping from flowers into nearby traps, creating a conflict partly offset by autonomous selfing that buffers female function while leaving male function vulnerable.

## INTRODUCTION

There is much interest in the potential for carnivorous plant trap leaves to ensnare their insect pollinators, because this might set up a pollinator–prey (p-p) conflict if the same species are in service as both pollinators and prey and the plants are dependent upon these pollinators for seed set. To establish that a true conflict exists, Jürgens *et al.* (2012) defined three preconditions: (1) the plant must be pollinator dependent; (2) there must be overlap between the pollinator and prey species; and (3) reproductive success must be pollen and/or pollinator limited (*sensu*Knight *et al.*, 2005). To date, clear evidence for such a conflict has been rare; for example, in *Pinguicula vallisneriifolia* (Lentibulariaceae), the greatest overlap in pollinator and prey was with thrips, a common floral visitor, and the micro-environment was an important determinant of the overlap (Zamora, 1999). Two systems have been described in *Drosera* where p-p conflicts could be avoided, which are not mutually exclusive: spatial separation of flowers from trap leaves (Anderson, 2010), and cue-partitioning, whereby visual signals (Joel *et al.*, 1985) or chemical signals (El-Sayed *et al.*, 2016) might partition pollinators away from trap leaves.

Tall flowering stems in *Drosera* that spatially separate basal leaves from flowers have been hypothesised as an evolutionary response that minimizes this pollinator–prey conflict (Givnish, 1989). Both Murza *et al.* (2006) and Anderson (2010) note that this hypothesis has been tested inadequately. For instance, Murza *et al.* (2006) studied *Drosera anglica*, a species with basal trap leaves and leafless scapes, and found evidence of only a minor p-p conflict, because distinct arrays of floral visitors and prey were observed. Likewise, Anderson (2010) compared floral visitors and leaf prey in *Drosera cistiflora*, which bears short scapes and both basal and cauline leaves, with *Drosera pauciflora*, which produces leafless long scapes atop basal leaves. No significant differences were found between these species in terms of their floral visitors, and only one leaf trap contained a floral visitor. The near absence of conflict in these spatially separated systems is consistent with the hypothesis that spatial separation of flowers and trap leaves reduces p-p conflict. Two features of long-scaped species are likely to contribute. First, the senescence of basal leaves prior to flowering provides a temporal separation of trap leaves and flowers, further reducing the opportunity for conflict (Bates, 1991). Second, long, leafless scapes may not provide a spatial arena for conflict even when basal leaves persist, given the height preferences of flying insects, such that short-scaped species might be exposed largely to a soil-surface fauna rather than the floral visitors occupying the higher ‘strata’ of forb communities. If spatial separation indeed abates conflict, then p-p conflict should be expected to be most likely and most intense in species in which flowers and trap leaves are closely juxtaposed, which is the condition that remains poorly documented.

Other mechanisms and floral architectures also require further investigation. El-Sayed *et al.* (2016) used a model with cauline leaf traps in close proximity to flowers (*Drosera auriculata*), where they found that floral odour attracted both pollinator and prey insect species, whereas trap odour attracted only prey insects. In their study, flowers were ∼4 cm from trap leaves, and thus small insects could potentially avoid leaf snares while visiting flowers. Furthermore, although the hypothesis that leaf traps might be attractive to prey species has received much attention, there has been little consideration that the production of flowers might increase both pollinator and non-pollinator prey (but see Salces-Castellano *et al.*, 2016). Tagawa *et al.* (2018*a*) found that although flowers on *Drosera makinoi* and *Drosera toyoakensis* were not needed to capture prey, they did find an exploitative relationship between *Drosera* spp. and other co-flowering species. In their study system, these species were not pollinator limited, were selfing species and were considered moderately inbreeding with flowers close to trap leaves (Tagawa *et al.*, 2018*a*, 2022). Despite the prediction that conflict should be most intense where flowers and traps are closely juxtaposed, p-p conflict has not, to our knowledge, been documented in such a species. We therefore identify two unresolved questions: whether a p-p conflict occurs in a species in which leaf snares are very close to flowers, the conditions in which conflict is predicted to be most intense (Jürgens *et al.*, 2012, 2015); and, separately, whether such capture arises because flowers attract pollinators that are then ensnared or because pollinators are captured accidentally during foraging.

The genus *Drosera* is a species-rich group comprising ∼248 species, with centres of diversity in Africa, Australia and South America. Commonly known as carnivorous sundews, they typically inhabit low-nutrient environments. To survive in these resource-poor habitats, their leaves (which can be basal or cauline) feature glandular hairs that produce adhesive, enzymatic secretions designed to ensnare and digest arthropods for supplemental nutrients. Their flowers are pedunculate and borne on short to long racemes (peduncles, scapes).

To address these questions, we required a species in which flowers and trap leaves are closely juxtaposed during flowering, such that the predicted high-conflict condition is met and the behaviour of insects at flowers and traps can be observed directly. We selected *Drosera hookeri* because it exhibits an unusually close juxtaposition of reproductive and carnivorous structures; we do not test the effect of flower–trap distance itself, which would require comparison among species differing in separation (cf. Anderson, 2010). This species features indeterminate flowering and tall scapes that bear a dense array of cauline leaf traps located centimetres from the open flowers. To test the p-p hypothesis in *D. hookeri*, we structured our study around the three preconditions established by Jürgens *et al.* (2012), alongside a specific test of the hypothesis that flowers facilitate pollinator capture. First, to evaluate pollinator dependence, we assessed the breeding system to determine the extent to which seed set requires pollinator-mediated pollen transfer, quantified as autonomous autofertility and its complement, pollinator dependence (*sensu*Lloyd and Schoen, 1992; Rodger and Ellis, 2016). Second, to test for limitations to reproductive success, we determined experimentally whether the system was pollen limited. Third, to establish the overlap in pollinator and prey, we estimated pollinator community abundance and scored leaf prey captures throughout the season. Finally, to test the hypothesis of floral facilitation of pollinator capture, we filmed insect behaviour at flowers and leaf traps and manipulated plants in a paired experiment to determine whether the presence of flowers directly increases the risk of trapping pollinators.

We predicted that close spatial proximity of flowers and trap leaves would create the potential for p-p conflict and that the consequences of this conflict would depend on the extent of pollinator-mediated reproduction, affecting female fitness through pollen receipt and male fitness through the loss of pollen vectors.

## MATERIALS AND METHODS

### Study sites

We conducted the studies on granite outcrops near Tingha (29°53′41″S, 151°17′42″E, 885 m a.s.l.) and the Copeton Dam (29°56′11.31′S, 151°02′08.45″E, 716 m a.s.l.), eastern Australia. These outcrops are ∼250 km long and are part of the ∼282-Myr-old Bundarra Granites (Phillips *et al.*, 2011) which are part of the extensive New England Batholith that occupies an area of 15 000 km^2^ in eastern Australia (Shaw and Flood, 1981). Vegetation on the outcrops is influenced by soil depth, with small forbs, such as *Drosera glanduligera*, *D. hookeri*, *Brachyscome stuartii* and *Wurmbea biglandulosa*, occurring as ephemeral assemblages during winter and spring on the shallow depressions in the granite. In the study sites, ephemeral plants emerge after winter and spring rains but are transient. Depending on soil moisture, the flowering season is short, from 1 to 4 weeks, and rarely longer. In dry years (e.g. 2013, 2017–2020) flowering was sparse or cut short, and some plants did not flower or even appear above ground. We thus used several field seasons between 2007 and 2021 to complete the investigations.

### Study species, *Drosera hookeri*

We studied the morphologically variable *Drosera hookeri* R.P.Gibson, B.J.Conn & Conran, which is one of five species in the *Drosera peltata* species complex (Gibson *et al.*, 2012).

*Drosera hookeri*, a tuberous herb found in seasonally moist habitats in eastern Australia, flowers between September and November in the study sites. Plants die back shortly after flowering and reappear the following year in July–August, depending on the prior rainfall. Plants possess a rosette of basal leaves and produce one or (rarely) more vertical peduncles (scapes) that reach a mean height of 22.69 cm [mean ± standard deviation (s.d.) 22.69 ± 6.86 cm, range 12–40 cm, *N* = 47 plants). Basal and cauline pedicellate leaves have glandular hairs that produce a sticky mucilage that immobilizes insects. Plants sometimes start out with red basal leaves, which change to green over the season and often become clogged with grit and twigs throughout the season. Vertical scapes serve as a central axis from which the cauline trap leaves and flowers emerge. The trap leaves grow directly from the scape on stout pedicels, whereas the flowers are borne on flimsy pedicels ≤2 cm in length. Plants often bear only a single flower at any one time, but as plants age and develop, or during favourable wet spring conditions, they can produce multiple flowers concurrently. At peak flowering, the cauline trap leaves are produced a few centimetres from the flowers (mean ± s.d. 2.56 ± 1.25 cm, *n* = 47). Flowers are 1.2–1.5 cm wide, pale pink, actinomorphic, pentamerous, bisexual, apparently odourless and nectarless. *Drosera hookeri* has five stamens and three free styles that are divided into many slender segments to give multi-branched styles. Flowers open for only a few hours, from ∼09:00 to 13:00 h Australian Eastern Standard Time, and individual flowers open for only a single day. On cloudy days, flowers may not open, whereas in sunny weather, plants may produce a new flower every day. Fruits are a loculicidal capsule with numerous (∼280) small black seeds.

### Pollinator dependence

Thirty-nine *D. hookeri* plants in rosette form (basal leaves only, diameter >2 cm), located ≥3 m apart, were haphazardly selected from Tingha in late July 2007 for a glasshouse experiment to assess the requirement for pollination. Plants were scooped from the ground to a depth of ∼10 cm, transferred into small pots, watered immediately and transferred to a glasshouse at the University of New England, Armidale, NSW, Australia. An additional 12 plants in the Tingha population were tagged and left undisturbed until flowering in order that the fruit set from open field pollination could be measured. Translocated plants were kept at a constant 22 °C under a 12 h light/dark regimen and hand watered every 2–3 days during the experiment. A frame of fine gauze (of 300 μm mesh) was placed over plants to prevent insects from visiting flowers. Pollination treatments were as follows: autogamy, where flowers were left un-manipulated to test for automatic self-pollination (*N* = 13 plants, *n* = 68 flowers); hand self-pollination, where pollen was transferred from the anthers to the stigmatic area of the same flower (*N* = 13, *n* = 34); and outcross hand pollination, where flowers received pollen from a flower on a different individual (*N* = 10 plants treated, of which 9 produced fruit and were analysed; *n* = 24 flowers). On occasion, some flowers were spent before a pollination treatment could be applied, and these were allocated to the autogamy treatment. Fine metal probes were used as pollen wands in the hand pollination treatments, and they were cleaned between treatments (in 100 % ethanol and dried). Spare flowers (*n* = 3) were allocated to an apomixis trial, in which all anthers were removed before the stamens had dehisced, then flowers were monitored for fruit set. Fruits from the treatments were harvested when they were plump and beginning to darken and dry, but before dehiscence. The 12 open-field plants at Tingha provided a single descriptive measure of natural fruit set in the field and were not used in estimating the breeding-system indices, because they differed from the glasshouse treatments in growing conditions and in pollination. Fruit-to-flower ratios on 12 plants (*n* = 56 flowers) at Tingha were measured in October 2007, and fruits were then harvested for seed count. Individual fruits were stored in labelled tubes and air-dried. Seeds were counted and weighed (50 seeds per fruit) using a Sartorius Micro MC1 balance (model MC50CE).

### Limitations to reproductive success

To determine whether plants were limited by pollen availability, we conducted a pollen supplementation experiment in the field. This was conducted in the Copeton population (from 24 September to 26 October 2021). Using 53 flowering plants, we randomly allocated 13 plants as the open control treatments (no pollen added and open to pollinators) and a further 13 plants to each of the closed control (no pollen added, bagged to exclude pollinators), open supplementation (*n* = 14, outcross pollen added and open to pollinators) and closed supplementation (outcross pollen added, bagged and closed to pollinators) treatments. One flower on each plant was used for the treatments, and each hand-pollinated flower received ∼300 pollen grains (approximating the number of ovules) from two or three donor flowers located ≥5 m away and applied to the stigmas of the treatment flower. Outcross pollen was applied before stamen recurving. Anthesis is completed within a day for *D. hookeri*, and exclusion bags were removed after flowers had closed in order that plants had similar access to light and water to the unbagged treatments. Fruits were harvested 3–4 weeks later and seeds counted. Pollen limitation was quantified from the contrast between open control and open pollen-supplemented flowers, the only pair differing solely in pollen receipt (Larson and Barrett, 2000). The bagged (pollinator-excluded) treatments were not used to estimate pollen limitation; the contrast between the bagged control and bagged supplemented treatments differed in both the addition and the type of pollen and was therefore treated only as a field estimate of pollinator dependence (*sensu*Lloyd and Schoen, 1992), not as a test of pollen limitation or of self-compatibility.

### Flower-visitor observations

To characterize the flower visitors of *D. hookeri*, we used video cameras and stationary observations at plants to record visitation to flowers over several years (2007, 2011, 2012, 2014–2016 and 2020) as part of a broader community pollinator survey. We deployed Sony Handycams (models HDR-XR160, HDR-PJ540 and FDR-AXP35 4K) at flowering plants for ≤4.5 h on sunny days. Stationary observations of 10 min duration were undertaken in 2007, when we had fewer video cameras available for deployment. We reviewed the footage from 93 plants (∼131 h) and recorded the behaviour of floral visitors at the flowers.

### Overlap in pollinator and prey

To quantify the extent to which *D. hookeri* shares its pollinator and prey species, we measured community pollinator abundance alongside leaf captures. The availability of pollinators and potential leaf visitors at Copeton was measured using pan traps (Campbell and Hanula, 2007) made from three plastic bowls painted with ultraviolet-bright fluorescent blue or yellow spray paint or left unpainted (white). Data were collected using six pan trap sets before, throughout and after the *D. hookeri* flowering across multiple years (2015, 2016, 2020 and 2021), resulting in 38 trapping days in total. Sets of pan traps were deployed on level ground within the immediate proximity of *D. hookeri*. Flowering was scored in quartiles (t0 = before, t1 = early, t2 = mid, t3 = late and t4 = after). Following closure of *D. hookeri* flowers, captured insects were stored in 70 % ethanol, and pollinator species were counted and compared against flowering phenology.

Concurrently, to determine whether leaf traps were more effective on plants that were flowering than non-flowering, we monitored prey captures over time on plants in three 1 m × 1 m plot areas at Tingha in September 2007. Fifty plants were selected haphazardly within each plot. The number of buds, flowers and closed flowers were scored on plants every 7–10 days. At the same time, leaf prey was collected from both basal and cauline leaf traps using forceps. This provided prey samples from before, during and after peak flowering. In the laboratory, we identified insects to order.

### Floral facilitation of pollinator capture

We set up a paired experiment to test the hypothesis that the presence of flowers increases the risk of trapping pollinators. On a sunny day at Copeton, we used plants in groups of three (*n* = 9 groups). In each trio, one plant had a fresh flower, one had a spent flower from the day before, and one plant had a scape with advanced buds, but not yet in flower. Plants were ≥50 cm apart and matched for height (20–25 cm), number of cauline trap leaves (10 or 11) and plant colour. All plants had existing leaf prey, which were left intact until after the experiment to avoid leaf damage. Deploying multiple video cameras, we filmed the plants for visitation to the flowers and the upper four or five cauline trap leaves for 150 min. After video monitoring, we counted the number of pollinator species (hoverflies and bees) in the top five cauline leaves and reviewed the videos to compare pollinator visitation to plants with and without flowers.

### Statistical analyses

In the glasshouse breeding system experiment (Experiment 1), pollination treatments were applied to all flowers on a given plant rather than randomized within plants. Individual flowers are therefore subsamples within plants, and the plant is the true unit of replication (*N* = 13 for autogamy, *N* = 13 for hand-self, *N* = 9 for hand-outcross, and *N* = 12 for open-field pollination). Accordingly, the mean seed count and mean mass of 50 seeds (hereafter 50-seed mass) per plant were calculated and used as the response variables in all analyses (Supplementary Data Table S1).

Treatment effects on plant-level mean seed count and 50-seed mass were each tested using one-way ANOVA followed by Tukey’s HSD *post hoc* comparisons. Assumptions of normality and homogeneity of variance were assessed using Shapiro–Wilk and Levene’s tests, respectively (Supplementary Data Table S2). Fruit set was analysed using a *χ*^2^ test. A generalized linear mixed-effects model analysis using all individual flower-level data, with plant identity as a random intercept, is presented in Supplementary Data Table S3 as a sensitivity check; conclusions were consistent with the plant-level analysis.

For the field pollen supplementation experiment (Experiment 2), seed set was compared among treatments using a Kruskal–Wallis test with pairwise Mann–Whitney *U*-tests. The 50-seed mass was compared using one-way ANOVA. The index of pollen limitation was calculated as PL = 1 − (seed set of open control flowers/seed set of open pollen-supplemented flowers), following Larson and Barrett (2000). The index of autofertility was calculated as IAF = seed set(autogamy)/seed set(outcross), and the index of self-compatibility as ISI = seed set(self)/seed set(outcross), following Lloyd and Schoen (1992). Pollinator dependence was estimated as PD = 1 − IAF (Rodger and Ellis, 2016). Pollinator abundance among triplet plants was tested using three, two-tailed paired *t*-tests, with the null hypothesis as H0: μ noflowers = μ flowers (test 1, with flowers and yet to flower; test 2, with flowers and flowered yesterday; and test 3, with flowers yesterday and yet to flower). All analyses were conducted in Python 3 using scipy (v.1.12) and statsmodels (v.0.14).

## RESULTS

### Pollinator dependence: autofertility and self-compatibility

No insects were found inside the glasshouse enclosure in the trap leaves or in the mesh covers, indicating that we had a pollinator-free experimental set-up in the glasshouse. *Drosera hookeri* sets seed automatically, without the aid of pollinators (Fig. 1A). Fruit set was uniformly high across all treatments and did not differ significantly (*χ*^2^ = 1.15, d.f. = 3, *P =* 0.765). None of the apomixis flowers developed into fruit, consistent with fertilization being required for seed production. The mechanism for autogamous pollen deposition involves the staminal filaments incurving shortly after anthesis to bring the anthers into contact with the stigmas (Fig. 2).

**Fig. 1.**
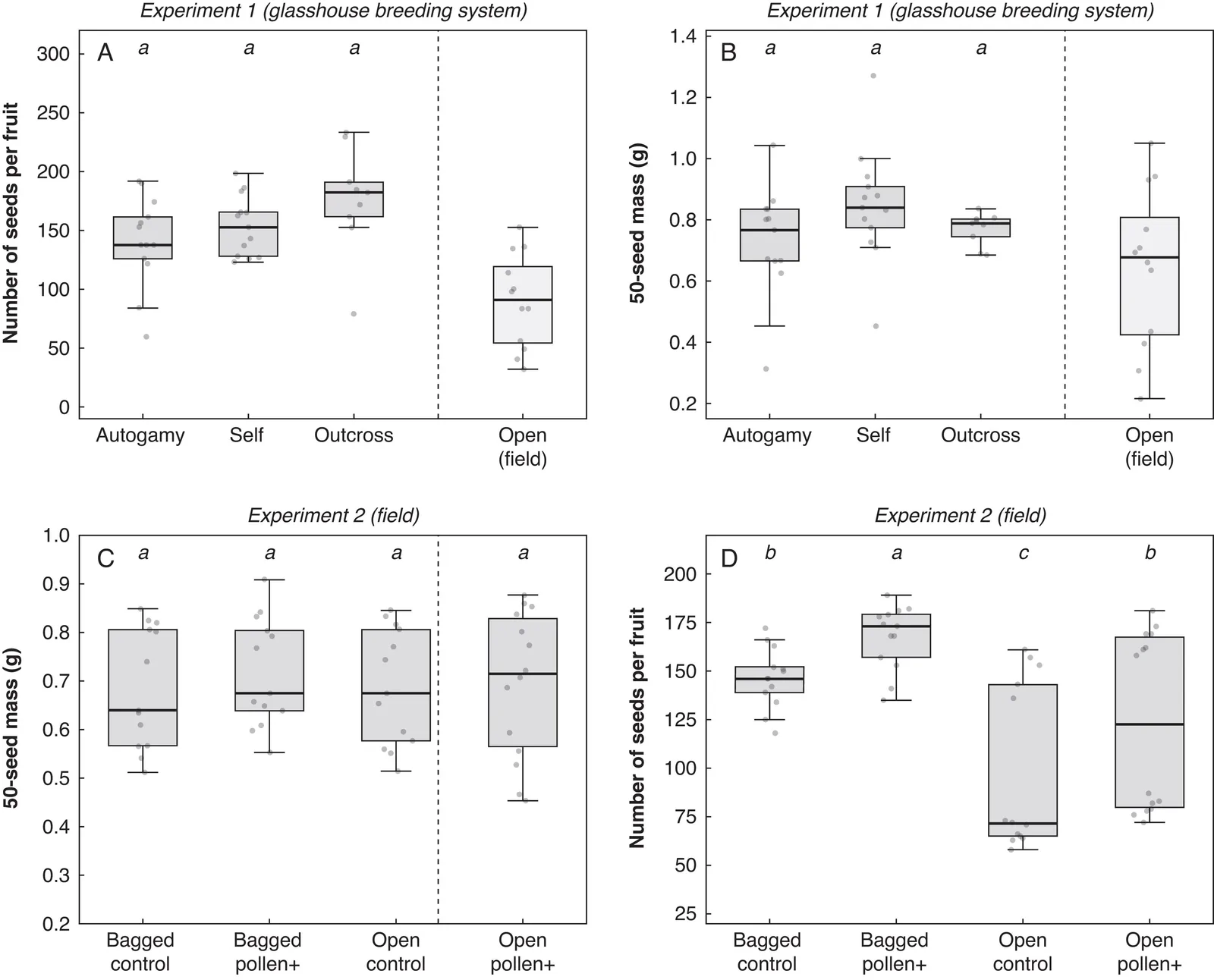
Breeding system and reproductive output of *Drosera hookeri*. (A, B) Seeds per fruit (A) and 50-seed mass (B) in the glasshouse breeding-system experiment, for autogamy (*N* = 13 plants), hand-self (*N* = 13) and hand-outcross (*N* = 9) pollination. Open-field pollination (*N* = 12) is shown for reference only and is excluded from the statistical comparison, because field and glasshouse conditions differed. (C, D) 50-seed mass (C) and seeds per fruit (D) in the field pollen-supplementation experiment (*n* = 13–14 flowers per treatment). In D, the open control versus open pollen-supplemented contrast quantifies pollen limitation; the bagged contrast is a field assessment of pollinator dependence, interpreted as pollen-quantity limitation of autonomous selfing rather than a benefit of outcross pollen. Boxplots show the median, interquartile range and 1.5 × interquartile range ; points are individual observations. Letters denote significant differences among glasshouse treatments in A, B (ANOVA, Tukey’s HSD test) and among treatments in D (Mann–Whitney *U*-test) at *P* < 0.05.

**Fig. 2.**
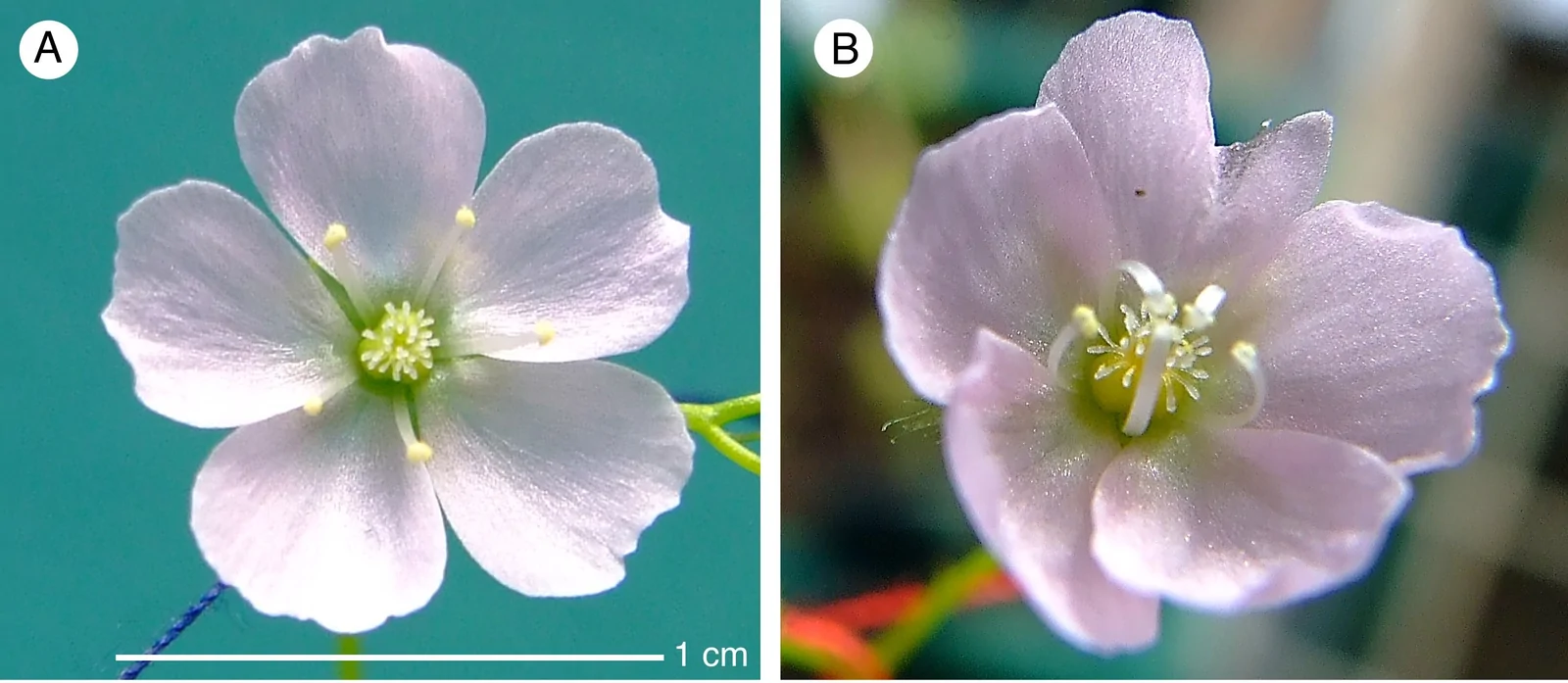
Flower of *Drosera hookeri* at anthesis (A) and 3 h later (B), when anthers have recurved towards the stigmas.

Among the three glasshouse pollination treatments, which differed only in the source of pollen, autogamous, hand-self and hand-outcross plants did not differ significantly in seeds per fruit (autogamy 140.8 ± 38.0, *N* = 13; hand-self 153.6 ± 25.4, *N* = 13; hand-outcross 176.0 ± 45.6, *N* = 9; one-way ANOVA across the three glasshouse treatments, all pairwise Tukey *P* > 0.05; Fig. 1A). Assumptions of normality (Shapiro–Wilk *W* = 0.951, *P =* 0.120) and homogeneity of variance (Levene’s *W* = 0.439, *P =* 0.648) were met. The absence of a difference between autogamous and hand-pollinated treatments indicates that seed production does not require pollinator-mediated pollen transfer, and the absence of a self-versus-outcross difference indicates strong self-compatibility. For reference, open-field plants at Tingha set fewer seeds per fruit (89.9 ± 39.8, *N* = 12); this value is reported descriptively only and is not compared statistically with the glasshouse treatments, because the settings differed in growing conditions and in pollination (Fig. 1A).

The mass of 50 seeds did not differ significantly among the glasshouse treatments when analysed at the plant level (one-way ANOVA: *F*_2,32_ = 2.02, *P =* 0.15; Fig. 1B). Results were unchanged using a non-parametric Kruskal–Wallis test (Supplementary Data Table S2). This result contrasts with the flower-level generalized linear mixed-effects model analysis (Supplementary Data Table S3), which detected a significant difference between hand-self and autogamous seeds (*P =* 0.023). The discrepancy reflects the inflated precision associated with treating non-independent flowers as replicates, and the plant-level analysis is considered the more appropriate test. The absence of a difference in seed mass between self and outcross treatments is consistent with strong self-compatibility and low inbreeding depression in this population.

The IAF was 0.80, indicating that *D. hookeri* achieves ∼80 % of its maximum seed set without pollinator intervention. Pollinator dependence was correspondingly low (PD = 0.20). The ISI was 0.87, confirming strong self-compatibility. In field conditions (the bagged-treatment contrast in Experiment 2, interpreted as a field estimate of pollinator dependence only), similar values were obtained (IAF = 0.87, PD = 0.13); this field shortfall is likely to reflect the pollen-quantity limitation of autonomous selfing identified below, rather than a requirement for pollinator-delivered outcross pollen (Supplementary Data Table S4).

### Pollen limitation

Plants were shown experimentally to be only weakly pollen limited in the Copeton population in 2021, on the basis of the contrast between open control and open pollen-supplemented flowers, which differ only in pollen receipt. The 50-seed mass did not differ significantly among the Experiment 2 treatments (one-way ANOVA *F*_3,49_ = 0.17, *P =* 0.917; Fig. 1C). For seed set, pollen supplementation to open flowers significantly increased seeds per fruit relative to open control flowers (pollen-supplemented 123.6 ± 46.1, *n* = 14; control 98.6 ± 42.9, *n* = 13; Mann–Whitney *U* = 38, *P =* 0.011; Fig. 1D), the contrast that quantifies pollen limitation. The index of pollen limitation for open flowers was weak (PL = 0.20). The bagged (pollinator-excluded) treatments are reported in Fig. 1D as a field assessment of pollinator dependence rather than of pollen limitation. Under pollinator exclusion, flowers receiving supplemental outcross pollen before autonomous self-deposition set significantly more seed than flowers relying on autonomous selfing alone (167.5 ± 16.5 vs. 146.5 ± 15.5 s.d., *n* = 13 each; *U* = 27, *P* = 0.004). Because the glasshouse hand-pollination treatments showed no difference between self and outcross pollen when pollen quantity was not limiting (Fig. 1A), this field increase is most likely to reflect pollen-quantity limitation of autonomous selfing rather than a quality advantage of outcross pollen.

### Flower visitors

Over the 6 years of video sampling (131.5 h of floral monitoring at 93 plants), where we monitored 96 flowers, 52 % received no floral visitors during anthesis (mean ± s.e.m., 0.47 ± 0.03 visitors per flower). However, in 2016, the best field season for flowering, most flowers received a visit (*n* = 38 flowers, 68 % visited), with an average of 2.72 ± 0.92 pollinators per hour. We recorded 381 floral visitors overall, with most visitations occurring between 09:00 and 10:00 h. In decreasing order of abundance, these visitors were: hoverflies, *Melangyna viridiceps* (Diptera, Syrphidae, *n* = 289) and *Simosyrphus grandicornis* (Diptera, Syrphidae, *n* = 26), a native bee, *Lasioglossum brunnesetum* (Hymenoptera, Halictidae *n* = 12), with the remaining as infrequent visitor types (Fig. 3A–D). *Melangyna viridiceps*, *Simosyrphus grandicornis* and *Lasioglossum brunnesetum* always contacted the anthers and stigma lobes while probing the flowers and were thus considered to be the primary pollinators. Time spent at flowers varied among visits but averaged ∼16 s and did not vary significantly among species.

**Fig. 3.**
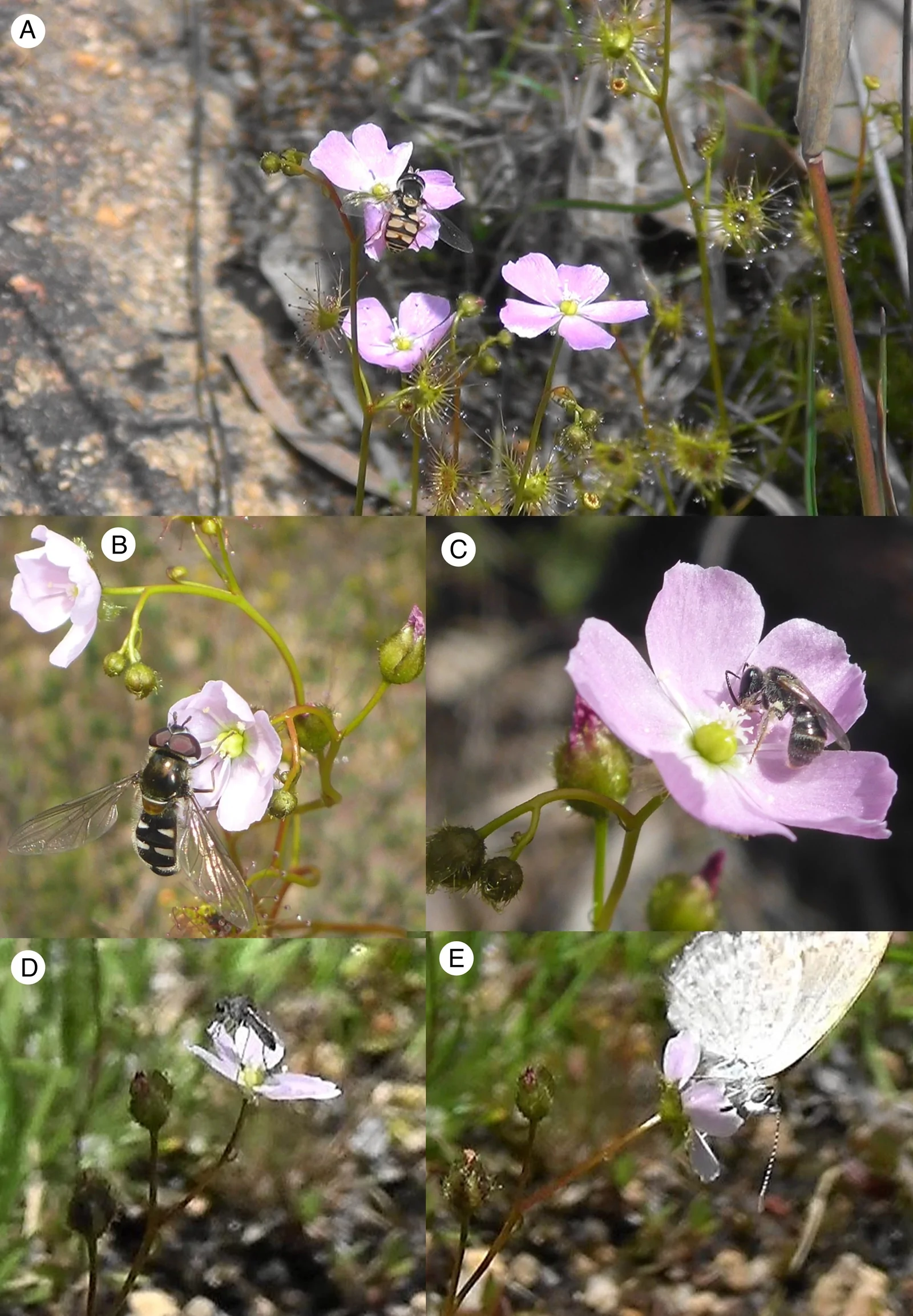
Floral visitors of *Drosera hookeri*. (A) *Simosyrphus grandicornis* (Diptera: Syrphidae). (B) *Melangyna viridiceps* (Diptera: Syrphidae). (C) *Lasioglossum brunnesetum* (Hymenoptera: Halictidae). (D) *Geron* sp. (Diptera: Bombyliidae). (E) *Zizina otis* (Lepidoptera: Lycaenidae). Note the close proximity of flowers and trap leaves in A. All images from CL Gross.

### Overlap in pollinator and prey

To contextualize the overlap between potential pollinators and prey, pan traps yielded a total of 421 arthropod individuals (∼86 species), including the identified pollinators in decreasing order of abundance: *Melangyna viridiceps* (16.86 %), *Lasioglossum brunnesetum* (6.65 %) and *Simosyrphus grandicornis* (1.43 %). When comparing pan trap colour, the hoverflies were more attracted to yellow pans, whereas for the native bee there was no colour preference. *Melangyna viridiceps* and *Lasioglossum brunnesetum* represented the first and fifth most abundant species caught in the pan traps. From these data, we deduce that pollinators were abundant at Copeton in the survey years and particularly during spring, when *D. hookeri* is in flower (Supplementary Data Fig. S1).

A total of 1246 arthropods were excised from the trap leaves (see examples in Fig. 4), and we were able to identify to order ∼72 % of specimens. The most common order found in the leaf traps was Diptera, followed by Hemiptera, Hymenoptera, Lepidoptera and Coleoptera (Fig. 5). Leaf prey was found in the traps over all time periods, with a peak in time period 4, which coincided with peak flowering in *Drosera hookeri* (Fig. 5). Within Diptera, the majority of captures occurred during peak flowering, and most were hoverflies, such as *Melangyna viridiceps* and *Simosyrphus grandicornis* (i.e. pollinators, Fig. 4). In contrast, for the Hemiptera, most captures were found in leaves prior to flowering (Fig. 5). The pollinator taxa (hoverflies and native bees) accounted for 57 % of identified leaf-trap specimens.

**Fig. 4.**
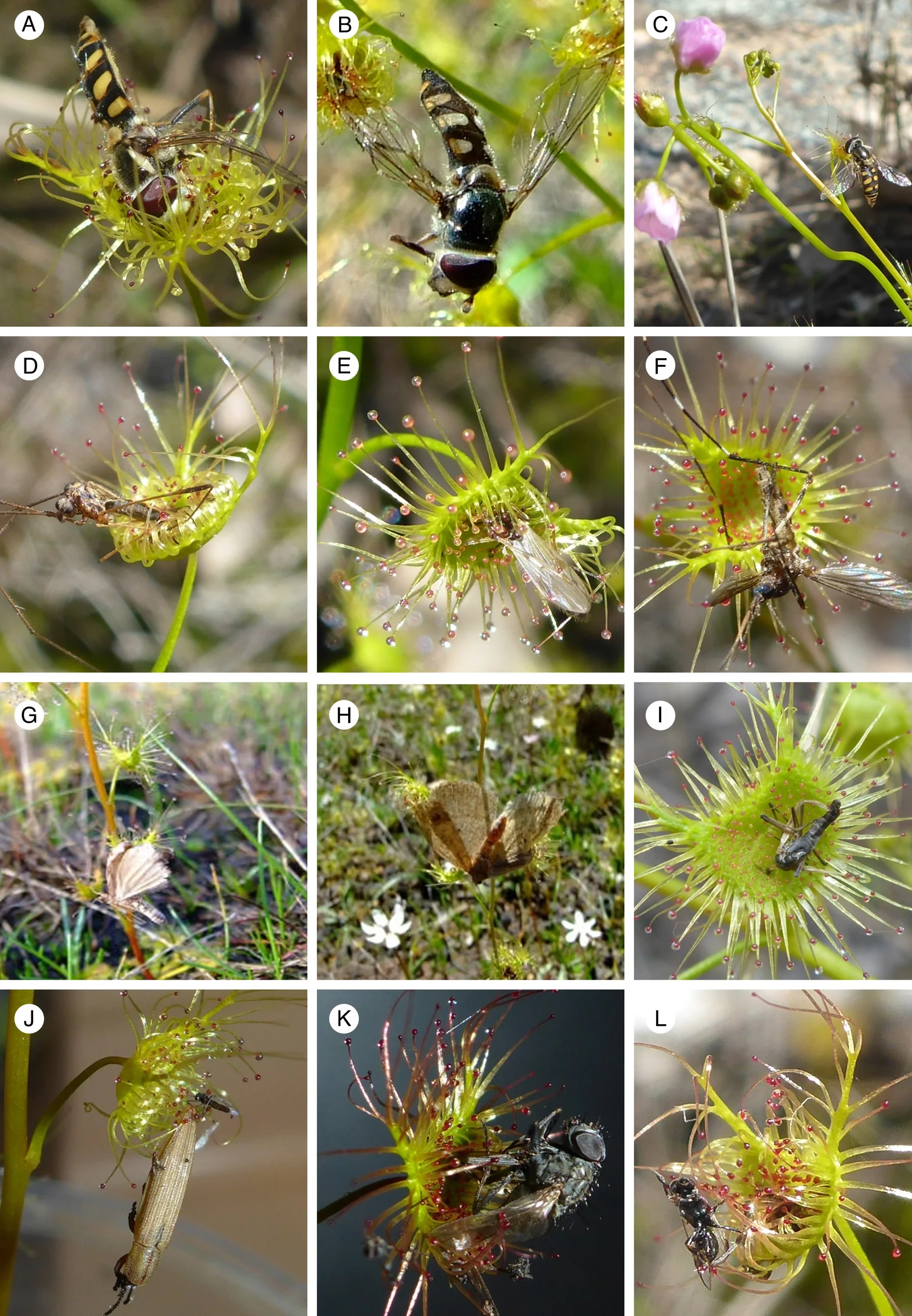
A selection of prey from *Drosera hookeri* leaves. (A–C) *Simosyrphus grandicornis* (Diptera: Syrphidae). (D–F) Crane flies (Diptera: Tipuloidea). (G) *Zizina otis* (Lepidoptera: Lycaenidae). (H) Noctuid moth (Lepidoptera: Noctuidae). (I) Crane fly (Diptera: Tipuloidea). (J) Click beetle (Coleoptera: Elateridae). (K) Muscoid fly (Diptera: Muscidae). (L) *Lasioglossum* sp. (Hymenoptera: Halictidae). Images from CL Gross and WA Waters.

**Fig. 5.**
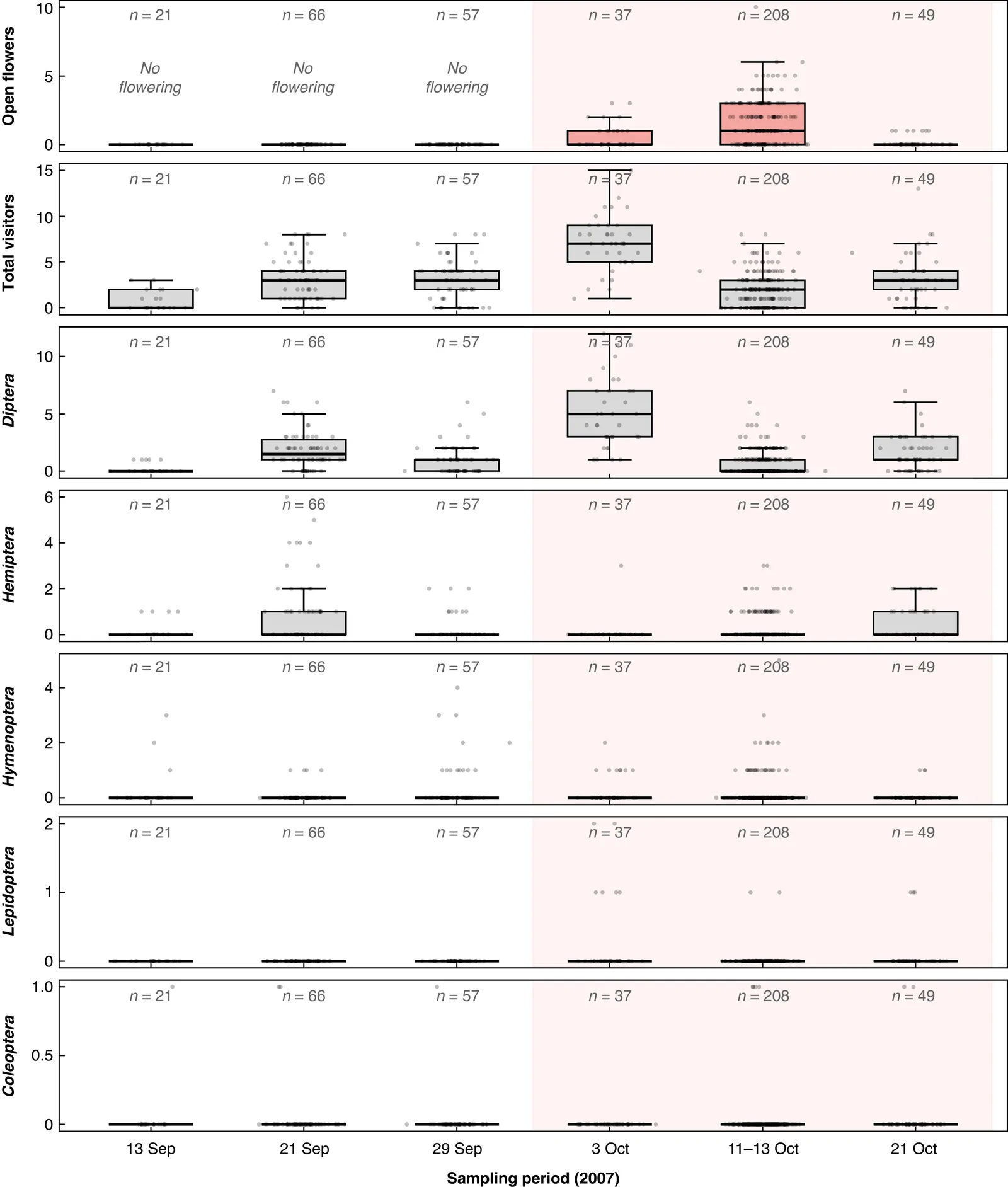
Prey captured by *Drosera hookeri* leaves across six sampling periods at Tingha in 2007. The top panel shows the number of open flowers per plant; no flowering occurred during the first three sampling periods. Subsequent panels show the total number of prey per plant and the breakdown by insect order: Diptera, Hemiptera, Hymenoptera, Lepidoptera and Coleoptera. Boxplots show the median (horizontal line), interquartile range (box) and 1.5 × interquartile range (whiskers); individual data points are overlaid. Sample sizes per period are shown at the top of each panel. The shaded region indicates the flowering period. Sampling dates in 2007 for each period are as follows: 1 = 13 September; 2 = 20–21 September; 3 = 29 September; 4 = 3–4 October; 5 = 10–13 October; and 6 = 21 October. Period 5 combines data from two consecutive census dates (11 and 13 October 2007).

### Floral facilitation of pollinator capture

From the paired experiment videos taken on a single day in 2014, we found that pollinators (*n* = 5; 3 hoverflies, *M. viridiceps*, 1 lycaenid butterfly *Zizina otis*, 1 *Geron* sp. Diptera Bombyliidae; Fig. 3D, E) visited only plants with open flowers, and they had no interest in the leaf traps, never visiting plants without flowers. Hoverflies were the only pollinators we found in leaf traps, and they were found only on plants that had flowers or spent flowers (in flower, 2.56 ± 0.65 hoverflies per plant, *n* = 9 plants; spent flowers, 2.89 ± 0.45 hoverflies per plant, *n* = 9 plants; no flowers, 0 hoverflies, *n* = 9 plants). Using a series of two-tailed paired *t*-tests of hoverfly captures, we rejected the null hypotheses that leaves on plants yet to flower had the same number of hoverflies as plants of the same size in flower (*t* = 3.94, d.f. = 8, *P =* 0.004) or with spent flowers (*t* = −6.35, d.f. = 8, *P =* 0.0002). In plants with a fresh flower, compared with plants with a spent flower, leaf traps did not have significantly different numbers of leaf prey (*t* = 0.47, d.f. = 8, *P =* 0.65).

The mechanism we observed by which pollinators end up being trapped in leaves appears to be accidental, with pollinators slipping or falling from the flower when it dips under their weight, bringing it onto or into close proximity to leaf traps. Flimsy pedicels or petals bend to bring the flowers into close contact with the trap leaves. Prior to capture, floral visitors generally foraged at the stigma and anthers (Fig. 3C) at the centre of the flower for pollen before transitioning to the petals to collect dehisced pollen, where loss of footing occasionally resulted in entrapment. On one of the videos taken in 2016, a bee slips from the flower into a trap and escapes after considerable struggle (Supplementary Data Video S1), made all the more difficult by the flower being pulled towards the leaf trap. Although we found no evidence of hoverflies or native bees being attracted to the leaf traps, we observed that hoverflies occasionally encounter the cauline leaves during flowering and appear to hesitate or dart away, suggesting a degree of trap aversion in flight.

## DISCUSSION

The results provide clear evidence that a p-p conflict exists in *D. hookeri*. Pollinators are not directly attracted to traps but are instead incidentally captured during foraging, and flowering is associated with increased capture of pollinators and other Diptera. However, we acknowledge that this correlation could also reflect seasonal background abundance of these insects rather than a direct effect of the flowers themselves. Nevertheless, this raises the possibility that flowers serve a dual function in both pollination and prey acquisition. The occurrence of long peduncles in carnivorous genera that pose no risk to their pollinators, such as *Utricularia*, suggests that peduncle elongation is driven by pollinator attraction rather than conflict avoidance alone (Anderson and Midgley, 2001).

Taken together, the findings align with the conditions outlined by Jürgens *et al.* (2012): *D. hookeri* is likely to remain pollinator dependent for male function, exhibits overlap between pollinators and prey, and shows some degree of pollen limitation. Although trapping pollinators might reduce opportunities for pollen export, this cost is likely to be offset by nutritional gains. Prey digestion in *Drosera* enhances photosynthesis and chlorophyll production (Pavlovič *et al.*, 2014), which might buffer plants against environmental stress, particularly drought.

### Pollinator dependence and reproductive strategy

*Drosera hookeri* is not obligately pollinator dependent, because it can set seed autonomously via incurving staminal filaments that bring anthers into contact with the stigmas. This occurs within 2–6 h of anthesis. After this, petals become flaccid and contort around the ovary, enclosing it. Although rapid flower closure in response to mechanical stimulation has been documented in other *Drosera* species (Tagawa *et al.*, 2018*c*), closure in *D. hookeri* appears to be regulated temporally rather than triggered by touch.

The breeding-system experiments show high autofertility (IAF = 0.80) and strong self-compatibility (ISI = 0.87), with no significant difference in seed count or seed mass between selfed and outcrossed treatments. We therefore find no measurable benefit of outcross pollen for female reproductive output in this population. The absence of a self-versus-outcross difference, together with the high ISI, is consistent with inbreeding depression having been largely purged in a predominantly selfing breeding system (Husband and Schemske, 1996), although we note that inbreeding depression can be elevated under drought stress (Cheptou *et al.*, 2000). Pollinator dependence for female function is correspondingly low; any dependence on pollinators is expected to operate through male function (outcross siring), an expectation we did not measure directly but which follows for predominantly selfing species (Barrett, 2003).

### Overlap in pollinator and prey

*Drosera hookeri* does not exploit distinct guilds of insects for pollinators and prey, as has been found for *D. anglica* (Murza *et al.*, 2006). Instead, flowers appear to attract pollinators, particularly hoverflies, which are subsequently captured by nearby trap leaves, especially during flowering. This overlap is facilitated by the close spatial proximity of traps and flowers, a condition predicted to increase conflict risk (Jürgens *et al.*, 2012, 2015).

The observations suggest that capture occurs primarily during foraging rather than during approach or departure. Floral visitors typically forage at reproductive structures before moving onto petals to collect loose pollen, where loss of footing or bending of the flower can result in entrapment. This contrasts with hypotheses that pollinators actively avoid traps or that trap morphology is optimized exclusively for smaller prey (Anderson and Midgley, 2001). Nonetheless, occasional avoidance behaviour by hoverflies indicates that some degree of trap detection may occur, potentially mediated by chemical cues. Similar leaf-aversion behaviour has been documented in hoverflies (*Sphaerophoria menthastri*) visiting *Drosera toyoakensis* (Tagawa *et al.*, 2018*b*) and might be associated with volatile cues emitted by trap leaves (El-Sayed *et al.*, 2016).

### Reproductive consequences and resource trade-offs

Although *D. hookeri* sets most of its seed autonomously, seed production in the field appears only weakly pollen limited, and pollen supplementation raised seed set only modestly in open flowers. Variation in seed mass is likely to reflect maternal resource allocation, with fewer seeds receiving greater investment (Paul-Victor and Turnbull, 2009).

Despite this, *D. hookeri* exhibits low pollinator dependence for female function (PD = 0.13–0.20) and only weak pollen limitation (PL = 0.20). Autogamy, facilitated by delayed self-pollination, provides effective reproductive assurance in variable environmental conditions, including drought and reduced pollinator activity. The occurrence of pseudo-cleistogamy (normally chasmogamous flowers that can behave cleistogamously), in unfavourable conditions further supports this strategy and has been observed in other *Drosera* species (Murza and Davis, 2003, 2005), often coinciding with low pollinator activity (Vicens and Bosch, 2000). More broadly, the capacity for autonomous selfing is widely recognized as a key mechanism of reproductive assurance in unpredictable environments (Lloyd, 1965; Eckert *et al.*, 2010; Cross *et al.*, 2020).

However, pollinators remain important for outcross pollen dispersal and thus for male function. Even highly autofertile species can benefit from pollinator visitation through increased siring success and the maintenance of genetic diversity (Barrett, 2003). In this context, the capture of pollinators by *D. hookeri* might impose a greater cost on male than female function, because female reproduction is largely buffered by autonomous selfing.

Outcrossing is promoted through delayed selfing and the tendency for only one flower to be open at a time, reducing opportunities for geitonogamy. At the same time, the species retains a highly effective autogamous back-up (IAF = 0.80), suggesting a strategy that balances reproductive assurance with opportunities for outcrossing. The level of pollinator visitation observed here might therefore be sufficient to limit inbreeding while also contributing to nutrient acquisition through occasional pollinator capture in this nutrient-poor environment. Primarily autonomous selfing is expected to buffer female function against variation in pollinator service, especially in a species with only one open flower at any time.

### Mechanisms linking pollination and prey capture

The balance between pollination and carnivory raises the question of how pollinators are captured despite this apparent functional separation. The primary pollinator, *Melangyna viridiceps*, is attracted to flowers but not to trap leaves, suggesting a degree of sensory partitioning. However, the flimsy pedicels of *D. hookeri* might override this separation by increasing the likelihood of pollinator capture during foraging.

By facilitating pollen receipt prior to capture, this mechanism might allow plants to retain any benefits of outcross pollen receipt for male function while gaining additional nutrients. Pollinator strength might also influence outcomes, because some individuals, particularly bees, are able to escape from traps.

The mechanical properties of pedicels and their potential plasticity in drought conditions warrant further investigation. Drought and dehydration can alter pedicel structure, hydraulic function and mechanical integrity (e.g. Lehmann *et al.*, 2019; see also Seguí *et al.*, 2018: table 1; and see Gibson *et al.*, 2012). More broadly, the observed behaviour of floral visitors moving onto petals to collect dehisced pollen prior to capture suggests a specific behavioural pathway linking pollination and carnivory. Quantifying the frequency of these events and their dependence on floral morphology would help to determine whether this represents an adaptive mechanism.

### Conclusion

Despite clear potential for conflict, *D. hookeri* appears to operate under a system in which pollination and carnivory are partly integrated rather than strictly antagonistic. Low pollinator dependence, combined with the nutritional benefits of prey capture, is likely to reduce the fitness costs of trapping pollinators. The conflict is therefore likely to asymmetric, with stronger effects on male function through the loss of pollen vectors, whereas female function is buffered by autonomous selfing. Behavioural and mechanical interactions between flowers and traps might facilitate this balance. Understanding how these dynamics shift across environmental gradients will be key to resolving the evolutionary stability of pollinator–prey interactions in carnivorous plants.

## Supplementary Material

mcag170_Supplementary_Data

## Data Availability

The data that support the findings of this study are stored by and available from the University of New England and provided as open access, under a CC-BY 4.0 licence. Data can be accessed from https://doi.org/10.25952/z8jk-jf44 linked at https://hdl.handle.net/1959.11/54516.

## SUPPLEMENTARY DATA

Supplementary data are available at *Annals of Botany* online and consist of the following. Video S1: *Lasioglossum brunnesetum* foraging on a flower of *Drosera hookeri.* Table S1: descriptive statistics for the glasshouse breeding system experiment (Experiment 1), summarized at the plant level. Table S2: one-way ANOVA and Tukey’s HSD pairwise comparisons on plant-level means for the three glasshouse pollination treatments of the breeding system experiment. Table S3: Flower-level linear mixed-effects model for Experiment 1. Table S4: breeding-system indices for *Drosera hookeri*, organized by the parameter each estimates. Figure S1: The abundance of the four main pollinators of D. hookeri in a pooled data set representing 38 pan trap sampling days from 29 August 2015 to 11 February 2022.
