# Supplementary material for: Fatal attraction: flowers lure pollinators as prey in the carnivorous Drosera hookeri (Droseraceae)
Source: Ann Bot. 2026 Jun 22;138(5):1511–22. doi: 10.1093/aob/mcag170 (PMC13715023; doi:10.1093/aob/mcag170)
Supplement: Supplementary file 1 [file mcag170_supplementary_data.zip › Tables S1-S4.docx]

**Supplementary Tables S1–S4**

*Gross et al., Drosera hookeri (AOB-2026-133-R2)*

**Table S1**. Descriptive statistics for the glasshouse breeding system experiment (Experiment 1), summarised at the plant level. Plant-level means were calculated by averaging across all flowers within each plant. N = number of plants (the unit of replication); n = total number of flowers measured. The three glasshouse treatments (autogamy, hand-self, hand-outcross) are those compared statistically (Table S2); the open-field treatment is included here as a descriptive reference only, as it differed from the glasshouse treatments in growing conditions as well as in pollination. (a) Number of seeds per fruit. (b) 50-seed mass (g).

***(a) Number of seeds per fruit***

| **Treatment** | **N (plants)** | **n (flowers)** | **Mean** | **SD** | **Median** | **Min** | **Max** |
| --- | --- | --- | --- | --- | --- | --- | --- |
| Autogamy | 13 | 68 | 140.8 | 38.0 | 137.8 | 59.5 | 191.7 |
| Self | 13 | 34 | 153.6 | 25.4 | 152.7 | 123.0 | 198.4 |
| Outcross | 9 | 24 | 176.0 | 45.6 | 182.0 | 79.0 | 233.0 |
| Open (field, descriptive) | 12 | 12 | 89.9 | 39.8 | 90.8 | 32.0 | 152.5 |

***(b) 50-seed mass (g)***

| **Treatment** | **N (plants)** | **n (flowers)** | **Mean** | **SD** | **Median** | **Min** | **Max** |
| --- | --- | --- | --- | --- | --- | --- | --- |
| Autogamy | 13 | 68 | 0.719 | 0.187 | 0.766 | 0.313 | 1.043 |
| Self | 13 | 34 | 0.847 | 0.186 | 0.839 | 0.453 | 1.271 |
| Outcross | 9 | 24 | 0.770 | 0.053 | 0.788 | 0.684 | 0.836 |
| Open (field, descriptive) | 12 | 12 | 0.645 | 0.263 | 0.677 | 0.215 | 1.050 |

**Table S2**. One-way ANOVA and Tukey HSD pairwise comparisons on plant-level means for the three glasshouse pollination treatments of the breeding system experiment (Experiment 1; N = 35 plants: 13 autogamy, 13 hand-self, 9 hand-outcross). The open-field treatment is excluded from this comparison because it differed from the glasshouse treatments in growing conditions as well as in pollination; it is reported descriptively in Table S1. Assumptions of normality (Shapiro-Wilk on residuals) and homogeneity of variance (Levene's test) are reported for each response. (a) Number of seeds per fruit. (b) 50-seed mass (g).

***(a) Response: Number of seeds per fruit***

| **Test** | **Statistic** | **df** | **P** |
| --- | --- | --- | --- |
| One-way ANOVA | F = 2.541 | 2, 32 | 0.095 |
| Shapiro-Wilk (residuals) | W = 0.951 |  | 0.120 |
| Levene (homogeneity) | W = 0.439 | 2, 32 | 0.648 |

***Tukey HSD pairwise comparisons***

| **Comparison** | **Mean difference** | **95% CI lower** | **95% CI upper** | **P (adjusted)** | **Significant** |
| --- | --- | --- | --- | --- | --- |
| Autogamy vs Outcross | 35.248 | -3.230 | 73.726 | 0.078 | No |
| Autogamy vs Self | 12.860 | -21.945 | 47.665 | 0.639 | No |
| Outcross vs Self | -22.388 | -60.866 | 16.090 | 0.338 | No |

***(b) Response: 50-seed mass (g)***

| **Test** | **Statistic** | **df** | **P** |
| --- | --- | --- | --- |
| One-way ANOVA | F = 2.017 | 2, 32 | 0.150 |
| Shapiro-Wilk (residuals) | W = 0.922 |  | 0.016 |
| Levene (homogeneity) | W = 2.105 | 2, 32 | 0.138 |

*Note: a marginal departure from normality was detected for 50-seed mass; a non-parametric Kruskal-Wallis test confirmed the non-significant result (H = 4.96, P = 0.084).*

***Tukey HSD pairwise comparisons***

| **Comparison** | **Mean difference** | **95% CI lower** | **95% CI upper** | **P (adjusted)** | **Significant** |
| --- | --- | --- | --- | --- | --- |
| Autogamy vs Outcross | 0.051 | -0.123 | 0.226 | 0.750 | No |
| Autogamy vs Self | 0.128 | -0.029 | 0.286 | 0.129 | No |
| Outcross vs Self | 0.077 | -0.098 | 0.251 | 0.532 | No |

**Table S3**. Flower-level linear mixed-effects model (GLMM) for Experiment 1 (pollination treatment as fixed effect, plant identity as random intercept; REML, N = 138 flowers from 47 plants; reference category autogamy), presented solely as a sensitivity check on the plant-level analysis in Table S2. (a) Number of seeds per fruit. (b) 50-seed mass (g).

***(a) Response: Number of seeds per fruit***

| **Fixed effect** | **Coefficient** | **SE** | **z** | **P** | **95% CI lower** | **95% CI upper** |
| --- | --- | --- | --- | --- | --- | --- |
| Intercept (Autogamy) | 149.826 | 7.852 | 19.082 | <0.001 | 134.437 | 165.215 |
| Open | -59.903 | 17.163 | -3.490 | <0.001 | -93.542 | -26.264 |
| Outcross | 29.603 | 13.907 | 2.129 | 0.033 | 2.346 | 56.861 |
| Self | 12.463 | 12.488 | 0.998 | 0.318 | -12.012 | 36.939 |
| Plant (intercept) variance | 216.202 |  |  |  |  |  |
| Residual variance | 2578.762 |  |  |  |  |  |

N observations = 138; N groups (plants) = 47; Log-likelihood = -727.538

***(b) Response: 50-seed mass (g)***

| **Fixed effect** | **Coefficient** | **SE** | **z** | **P** | **95% CI lower** | **95% CI upper** |
| --- | --- | --- | --- | --- | --- | --- |
| Intercept (Autogamy) | 0.746 | 0.027 | 27.555 | <0.001 | 0.693 | 0.799 |
| Open | -0.101 | 0.062 | -1.634 | 0.102 | -0.221 | 0.020 |
| Outcross | 0.026 | 0.050 | 0.521 | 0.603 | -0.071 | 0.123 |
| Self | 0.101 | 0.045 | 2.268 | 0.023 | 0.014 | 0.188 |
| Plant (intercept) variance | 0.002 |  |  |  |  |  |
| Residual variance | 0.034 |  |  |  |  |  |

N observations = 138; N groups (plants) = 47; Log-likelihood = 25.421

*Note: the GLMM treats individual flowers as replicates although treatments were applied at the plant level, and therefore overstates precision relative to the plant-level analysis; it detects a self-versus-autogamy difference in 50-seed mass (P = 0.023) that is not significant at the plant level (Table S2; Tukey P = 0.129).*

**Table S4**. Breeding-system indices for Drosera hookeri, organised by the parameter each estimates. Each index is derived from the single relevant contrast. IAF = Index of Autofertility (Lloyd and Schoen, 1992); ISI = Index of Self-Compatibility (Lloyd, 1965); PD = Pollinator Dependence = 1 - IAF (Rodger and Ellis, 2016); PL = Index of Pollen Limitation (Larson and Barrett, 2000). Glasshouse indices use plant-level mean seed counts (Experiment 1); field indices use flower-level seed counts (Experiment 2). The field IAF/PD (bagged contrast) is interpreted as a field estimate of pollinator dependence only; because the bagged contrast confounds pollen addition with pollen type it does not estimate pollen limitation or self-compatibility.

***Indices by concept***

| **Concept** | **Index** | **Formula** | **Value** | **Source experiment** |
| --- | --- | --- | --- | --- |
| Autofertility | IAF | autogamy / outcross | 0.800 | Exp 1 (glasshouse) |
| Pollinator dependence | PD | 1 - IAF | 0.200 | Exp 1 (glasshouse) |
| Self-compatibility | ISI | self / outcross | 0.873 | Exp 1 (glasshouse) |
| Pollinator dependence (field) | IAF / PD | bagged ctrl / bagged supp | 0.874 / 0.126 | Exp 2 (field, bagged) |
| Pollen limitation | PL | 1 - (open ctrl / open supp) | 0.202 | Exp 2 (field, open) |

***Treatment means used (Experiment 1, plant-level)***

|  | **Autogamy** | **Self** | **Outcross** |
| --- | --- | --- | --- |
| Mean seed count | 140.8 | 153.6 | 176.0 |
| N plants | 13 | 13 | 9 |

***Treatment means used (Experiment 2, flower-level)***

|  | **Bagged ctrl** | **Bagged supp** | **Open ctrl** | **Open supp** |
| --- | --- | --- | --- | --- |
| Mean seed count | 146.5 | 167.5 | 98.6 | 123.6 |
| N flowers | 13 | 13 | 13 | 14 |
